# Population-Adjusted Indirect Treatment Comparisons of Repotrectinib Among Patients with *ROS1+* NSCLC

**DOI:** 10.3390/cancers17050748

**Published:** 2025-02-22

**Authors:** Jürgen Wolf, Sarah Goring, Adam Lee, Byoung Chul Cho, Alexander Drilon, Yong Yuan, Dieter Ayers, Greta Lozano-Ortega, Ellen E. Korol, Sarah G. Korpach, Madeleine Crabtree, Lavanya Huria, Christophe Y. Calvet, D. Ross Camidge

**Affiliations:** 1 Center for Integrated Oncology, University Hospital of Cologne, 50937 Cologne, Germany; juergen.wolf@uk-koeln.de; 2 Broadstreet HEOR, Vancouver, BC V5Y 1L8, Canadaglozano-ortega@broadstreetheor.com (G.L.-O.); ekorol@broadstreetheor.com (E.E.K.); skorpach@broadstreetheor.com (S.G.K.); mcrabtree@broadstreetheor.com (M.C.); lhuria@broadstreetheor.com (L.H.); 3 Bristol Myers Squibb, Uxbridge UB8 1DH, UK; 4 Yonsei Cancer Center, Yonsei University College of Medicine, Seoul 03722, Republic of Korea; 5 Memorial Sloan Kettering Cancer Center, Weill Cornell Medical College, New York, NY 10065, USA; 6 Bristol Myers Squibb, Lawrenceville, NJ 08648, USA; 7 University of Colorado Cancer Center, Aurora, CO 80045, USA

**Keywords:** *ROS1* fusion-positive, lung cancer, population-adjusted indirect comparisons, repotrectinib, crizotinib, entrectinib, efficacy

## Abstract

Background: Head-to-head evidence comparing repotrectinib against other approved ROS1 tyrosine kinase inhibitors (TKIs) is not currently available. The objective of this study was to indirectly compare progression-free survival (PFS), the objective response rate (ORR), and the duration of response (DoR) for repotrectinib vs. crizotinib and vs. entrectinib in patients with TKI-naïve *ROS1+* locally advanced or metastatic non-small-cell lung cancer (aNSCLC). Methods: Using evidence from a systematic literature review, unanchored matching-adjusted indirect comparisons (MAICs) were used to estimate population-adjusted hazard ratios (HRs) for PFS and DoR and odds ratios (ORs) for ORR for repotrectinib vs. crizotinib and vs. entrectinib among patients with TKI-naïve aNSCLC. The MAICs were adjusted for imbalances in baseline patient characteristics that were pre-specified as being prognostic or predictive of treatment effects. Weighted Cox (for PFS and DoR) and logistic (for ORR) regression models were fit. Supplementary analyses (SAs) explored the impact of missing data and modeling assumptions on effect estimates. Results: The evidence base was formed by TRIDENT-1 EXP-1 (repotrectinib; N = 71), a pooled set of five trials involving crizotinib (N = 273), and the pooled ALKA-372–001/STARTRK-1 and -2 trials (entrectinib; N = 168). After population adjustment, repotrectinib was associated with statistically significant improvements in PFS relative to crizotinib (HR = 0.44; 95% confidence interval [CI]: 0.29, 0.67) and entrectinib (HR = 0.57; 95% CI: 0.36, 0.91). Differences in ORR and DoR were not statistically significant but numerically favored repotrectinib. SAs were consistent with the main analyses across all comparisons. Conclusions: The analysis demonstrated the strong benefits of repotrectinib in PFS, which was robust across different SAs and supported by numerically favorable results for DoR (where available) and ORR. These results, alongside the published TRIDENT-1 clinical data, further support repotrectinib as a potential new standard of care for TKI-naïve patients with *ROS1+* aNSCLC.

## Simple Summary

Repotrectinib, crizotinib, and entrectinib are tyrosine kinase inhibitors approved for *ROS1*-positive advanced non-small-cell lung cancer. Comparing the efficacy of these treatments is crucial for guiding decision-making; however, randomized head-to-head clinical trials involving repotrectinib are unavailable. This study indirectly compared these drugs, accounting for differences in patient populations across trials, and found that repotrectinib offers a statistically significant progression-free survival advantage over both crizotinib and entrectinib. Repotrectinib was also associated with a numerically higher objective response compared to both crizotinib and entrectinib and a numerically longer duration of response compared to entrectinib. These findings provide valuable insights to help make informed treatment recommendations for patients with *ROS1*-positive advanced non-small-cell lung cancer.

## 1. Introduction

*ROS* proto-oncogene 1 (*ROS1*) fusions are oncogenic drivers that have been identified in around 2% of patients with non-small-cell lung cancer and are more prevalent in non-squamous tumor types, younger individuals, female patients, and those without a history of smoking [1,2,3,4,5]. Tyrosine kinase inhibitors (TKIs) targeting *ROS1* fusions (*ROS1+*) are currently approved and recommended as the preferred first-line options in clinical guidelines for treating *ROS1+* locally advanced or metastatic non-small-cell lung cancer (aNSCLC) [6,7,8,9,10,11,12]. Despite high response rates with the early generation ROS1 TKIs crizotinib and entrectinib [13,14], secondary *ROS1* resistance mutations often emerge during treatment [15,16]. Furthermore, efficacy among patients with brain and central nervous system (CNS) metastases has been limited, with only short-lasting intracranial responses, if any [17,18]. Repotrectinib is a newer ROS1 TKI that is considered less susceptible to *ROS1* resistance mechanisms among patients with *ROS1+* aNSCLC and more active in the CNS; deep reductions in tumor volume and durable clinical activity have been observed, including in brain lesions [19].

The scarcity of *ROS1* fusions makes patient recruitment for clinical trials a challenge. To date, clinical trials assessing ROS1 TKIs have employed non-comparative study designs, such as single-arm trials. Repotrectinib was granted approval for adult patients with *ROS1+* aNSCLC based on results from the non-comparative TRIDENT-1 trial [20,21]; however, there are currently no randomized head-to-head clinical trials of repotrectinib against the early generation ROS1 TKIs.

Establishing the relative efficacy and safety of therapies is necessary to support decision-making. The objective of this study was to assess the comparative efficacy (progression-free survival [PFS], objective response rates [ORRs] and duration of response [DoR]) of repotrectinib vs. crizotinib and vs. entrectinib for the treatment of TKI-naïve patients with *ROS1+* aNSCLC. A secondary objective was to summarize the safety profiles of repotrectinib, crizotinib, and entrectinib.

## 2. Materials and Methods

### 2.1. Study Design

The comparative efficacy of repotrectinib relative to crizotinib and entrectinib was assessed using population-adjusted indirect comparisons, which are a widely adopted and preferred approach for indirectly comparing regimens that have not been tested in head-to-head trials [22,23]. For the current analysis, an unanchored matching-adjusted indirect comparison (MAIC) was implemented, in which individual patient data (IPD) from the non-comparative TRIDENT-1 trial were weighted in order to balance the patient characteristics among the non-comparative trials for crizotinib and entrectinib (for which only aggregate-level data are available) [24].

### 2.2. Evidence Base

The clinical trials informing the analysis were identified through a systematic literature review (SLR) according to pre-specified PICOS criteria, current to 11 July 2023 (see Table S1).

When multiple trials for a given comparator (crizotinib or entrectinib) were available, outcomes and patient characteristics were combined using weighted averages (by sample size) to create a pooled set of trials.

### 2.3. Adjustment Factors

Patient characteristics identified as being prognostic or predictive of treatment effect were identified a priori through a targeted literature review and confirmed with clinical experts [18,25,26,27,28,29,30,31,32,33,34,35]. Key characteristics were age, sex, race, Eastern Cooperative Oncology Group performance status (ECOG PS), smoking status, and presence of baseline CNS/brain metastasis. Additionally, the number of prior lines of therapy was identified but considered weakly predictive of outcomes in ROS1 TKI-naïve populations due to inconclusive evidence in *ROS1+* aNSCLC [18,25,27,33]. *TP53* mutation status was also considered prognostic but was not included due to non-reporting in trials. Due to potentially enhanced intracranial activity associated with repotrectinib compared with crizotinib and entrectinib, the presence of CNS/brain metastasis at baseline was identified as having an impact on the relative effect (e.g., hazard ratio [HR]) between these agents (termed an “effect modifier”). This means that estimated relative treatment effects between repotrectinib and crizotinib or entrectinib are expected to differ depending on the prevalence of CNS/brain metastasis at baseline.

### 2.4. Base Case Model Specification

In line with recommendations for conducting unanchored MAICs, the base case models were designed to adjust for all available prognostic and effect modifying factors [36]. Missing data on smoking status in TRIDENT-1 was addressed via imputation.

For the MAIC between repotrectinib vs. crizotinib, all available prognostic and effect modifying factors were considered as adjustment factors in the base case model, except for the number of prior lines of therapy. This factor was not included due to the a priori assumption that it may be weakly predictive of outcomes in ROS1 TKI-naïve populations, coupled with an initial assessment of large imbalances between the TRIDENT-1 population and the crizotinib trial populations, which would result in a substantial loss in precision. Baseline CNS/brain metastasis was not reported in one of the crizotinib trials (PROFILE 1001); this factor was set to 0% in the base case.

For the MAIC of repotrectinib vs. entrectinib, the base case analysis was adjusted for all available prognostic and effect modifying factors, including the number of prior lines of chemo-/immunotherapy.

### 2.5. Supplemental Analyses

Supplemental analyses (SAs) evaluated the impact of missing data and other modeling assumptions on effect estimates (see Tables S2 and S3). The variables explored in the SAs included CNS/brain metastasis at baseline, ECOG PS, smoking status, age, race, sex, and the number of prior lines of therapy. Additional SAs involved assessing residual bias to quantify the impact of missing data and non-overlapping eligibility for prognostic or predictive baseline patient characteristics.

Finally, a comparison with crizotinib solely informed by the registrational PROFILE 1001 trial (rather than the crizotinib All-evidence analysis set) was conducted.

### 2.6. Statistical Analysis

For the time-to-event outcomes PFS and DoR, Kaplan–Meier curves from comparator trials were digitized (DigitizeIt v2.5.9) to produce pseudo-IPD [37]. Weighted Cox proportional hazards models were fit (using MAIC weights), and relative effect estimates were generated as adjusted HRs, with 95% confidence intervals (CIs). For DoR, weighted Cox models were run only among the subset of responders, using weights derived from the full population. For ORR, weighted binomial generalized linear models were fit, and relative effect estimates were generated as adjusted odds ratios (ORs), with 95% CIs. Robust sandwich estimators were used to account for the additional uncertainty associated with the estimation of the weights for all outcomes. Each analysis was performed using the weighted and unweighted data from TRIDENT-1 EXP-1, providing both adjusted and crude (unadjusted) effect estimates.

The distribution of MAIC weights was examined; extreme weights that may have had a large influence on the estimated outcomes were identified. The effective sample size (ESS) was calculated to estimate the sample size that would be required to achieve the same level of precision as the weighted sample. The small ESS relative to the original sample size reflects highly variable weights due to poor overlap in patient characteristics [24,36].

The analysis was conducted in R version 4.2.2.

### 2.7. Safety

A formal comparison of safety was not pursued due to the inconsistent definition and reporting of treatment-related adverse events (TRAEs). Instead, a tabular summary of reported TRAEs for repotrectinib, crizotinib, and entrectinib was presented.

## 3. Results

### 3.1. Evidence Base

Eight relevant trials were identified in the SLR and included in the MAIC. The flow of trials through the SLR process is shown in Figure S1.

The evidence base for repotrectinib was informed using the IPD of the TKI-naïve cohort of TRIDENT-1 (EXP-1; N = 71).

Five trials assessed the efficacy of crizotinib and were pooled to form the “All-evidence” set: the registrational PROFILE 1001 trial (N = 53) [13], OO-1201 (N = 127) [38], AcSé (N = 37) [39], METROS (N = 26) [40,41], and EUCROSS (N = 34) [42]. Study geography and trial eligibility differed slightly relative to TRIDENT-1; notably, patients with ECOG PS 2 were eligible for the AcSé, EUCROSS, and METROS trials but were ineligible for TRIDENT-1 (see Table S4).

Three trials (ALKA-372-001, STARTRK-1, and STARTRK-2) assessed the efficacy of entrectinib. These trials (ALKA-STARTRK-1 and -2; N = 168) were pooled by the manufacturer prior to publication, using IPD [14]. Study locations and eligibility criteria were similar between TRIDENT-1 EXP-1 and ALKA-STARTRK-1 and -2 (see Table S4); however, patients with ECOG PS 2 were eligible for inclusion in ALKA-STARTRK-1 and 2.

A summary of baseline characteristics and relevant outcomes from all trials is provided in Tables S5 and S6.

### 3.2. MAIC Results for Repotrectinib vs. Crizotinib

The baseline characteristics in the crizotinib All-evidence analysis set were reasonably well aligned with TRIDENT-1 EXP-1, although the crizotinib All-evidence analysis set included about half as many patients with CNS/brain metastasis compared with TRIDENT-1 EXP-1 (Table 1). The proportion of patients with zero prior lines of therapy was also substantially lower in the crizotinib All-evidence analysis set (14%) relative to TRIDENT-1 EXP-1 (72%; Table 1). After applying MAIC weights, the baseline characteristics included for adjustment were well aligned. The ESS for TRIDENT-1 EXP-1 was 62.3 for PFS and ORR, representing a 12% reduction relative to the original sample size of 71.

**Figure 1:**
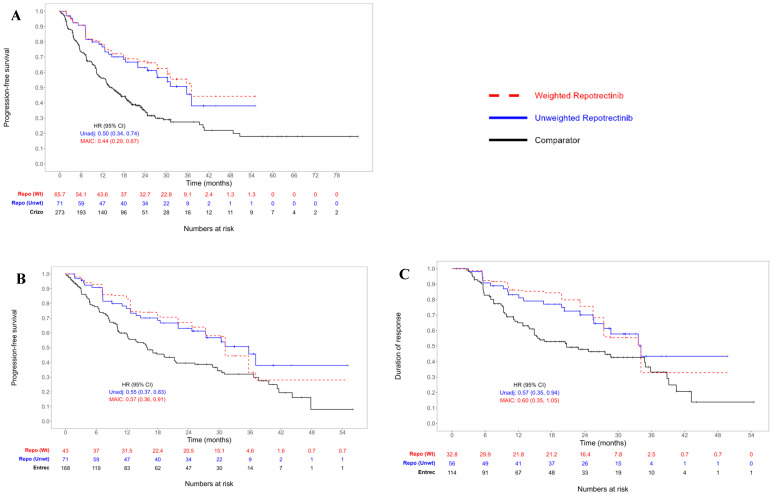
Kaplan–Meier curves of time-to-event outcomes: progression-free survival for repotrectinib vs. (**A**) crizotinib All-evidence analysis set and (**B**) entrectinib; (**C**) duration of response for repotrectinib vs. entrectinib. Abbreviations: Crizo—crizotinib; Entrec—entrectinib; Repo—repotrectinib; unadj—unadjusted; unwt—unweighted; wt—weighted. Note: The weighted n-at-risk is represented by the sum of the patient weights and is not equal to the ESS. The hazard ratios presented (both weighted and unweighted) are for repotrectinib vs. comparator. Hazard ratios < 1 favor repotrectinib.

Repotrectinib was associated with statistically significant improvements in PFS relative to crizotinib (MAIC HR = 0.44; 95% CI: 0.29, 0.67; Figure 1A). The MAIC-weighted median PFS for repotrectinib was 37.1 months (95% CI: 30.4, not estimable [NE]), compared with a median of 14.6 months (95% CI: 12.8, 18.5) in the crizotinib All-evidence analysis set (Table 2).

The MAIC-weighted ORR was numerically higher for repotrectinib (78%) compared with crizotinib (71%); the weighted OR favored repotrectinib, although the 95% CIs were wide and spanned the null value (MAIC OR = 1.48; 95% CI: 0.76, 2.87; Table 3).

A comparative analysis of DoR was not conducted due to a lack of Kaplan–Meier curves reported across the crizotinib trials (reported in none of the five trials). The median values in the four crizotinib trials that reported a median DoR ranged from 19.0 to 24.7 months (see Table S6), compared with 34.1 months (95% CI: 27.4, NE) in TRIDENT-1 EXP-1.

The SAs were broadly consistent with the main analyses (see Appendix S1, Figures S2 and S3); the estimates involving data from PROFILE 1001 only were associated with greater uncertainty (Figure S4). Residual bias assessments suggested the findings to be robust to missing and non-overlapping patient characteristics.

### 3.3. MAIC Results for Repotrectinib vs. Entrectinib

Most baseline characteristics were similar between TRIDENT-1 EXP-1 and ALKA-STARTRK-1 and -2; however, there were notable differences in the variables prior use of chemo- and/or immunotherapy and Asian race (Table 1). The weighted baseline characteristics were well aligned. The ESS for TRIDENT-1 EXP-1 was 30.1 for PFS and ORR (a 58% reduction relative to the original sample size of 71) and 21.5 for DoR (a 62% reduction relative to the sample size of 56 for responders).

Repotrectinib was associated with a statistically significant improvement in PFS relative to entrectinib (MAIC PFS HR = 0.57; 95% CI: 0.36, 0.91; Figure 1B). The MAIC-weighted median PFS for repotrectinib was 31.1 months (95% CI: 24.6, NE), compared with a median of 15.7 months (95% CI: 12.0, 21.1) for entrectinib (Table 2).

The MAIC-weighted ORR was numerically higher with repotrectinib (76%) compared with entrectinib (68%); the weighted OR favored repotrectinib, although the 95% CIs were wide and spanned the null value (MAIC OR = 1.53; 95% CI: 0.63, 3.66; Table 3).

The adjusted DoR numerically favored repotrectinib relative to entrectinib; however, the 95% CI spanned the null value (MAIC HR = 0.60; 95% CI: 0.35, 1.05, Figure 1C). The MAIC-adjusted median DoR for repotrectinib was 33.6 months (95% CI: 27.4, NE), compared with a median of 20.5 months (95% CI: 14.8, 34.8 Table 2).

Across all analyses, the SAs were largely consistent with the base case analysis and maintained the same direction of effect (see Appendix S1, Figures S5–S7).

### 3.4. Safety

Safety outcomes associated with repotrectinib, crizotinib and entrectinib are presented in the Table S7. Comparisons of safety outcomes should be made with caution due to differences across patient populations, study designs, and safety definitions.

## 4. Discussion

### 4.1. Summary of Findings and Clinical Context

Repotrectinib has demonstrated strong intracranial activity and durable intracranial response in pre-clinical and clinical trials [19,43,44]. In current clinical guidelines, both entrectinib and repotrectinib are preferred over crizotinib for patients with brain metastasis; crizotinib has demonstrated limited ability to cross the blood–brain barrier and a high incidence of new brain metastases during treatment [6,12,17,19]. The unanchored population-adjusted indirect comparisons in the current study—which addressed differences in prognostic and effect modifying factors between patients in the different trials—demonstrated that among TKI-naïve patients with *ROS1+* aNSCLC, repotrectinib was associated with about half the risk of progression or death compared with both entrectinib and crizotinib. These findings were robust to a range of sensitivity analyses. Differences in ORR (vs. entrectinib and vs. crizotinib) and in DoR (entrectinib only, due to non-reporting for crizotinib) showed a trend toward more favorable outcomes with repotrectinib.

The relative effect estimates represent population averages and are expected to generalize to TKI-naïve *ROS1+* aNSCLC populations that have around 30% of patients with asymptomatic CNS/brain metastasis at baseline per BICR for comparisons between repotrectinib and entrectinib and to populations comprising about 16% of patients with CNS/brain metastasis at baseline for comparisons between repotrectinib and crizotinib.

Population-adjusted indirect comparisons of intracranial outcomes were not conducted in the current study due to the small sample sizes among brain metastasis subgroups (N = 9 in TRIDENT-1), the non-reporting of brain metastasis-specific baseline characteristics in ALKA-STARTRK-1 and -2, and the non-reporting of intracranial outcomes for crizotinib. Hence, further efforts are needed to better understand the relative efficacy of repotrectinib and earlier generation ROS1 TKIs for intracranial outcomes.

The development of resistant mutations represents a key challenge of treatment with ROS1 TKIs [19,43,44,45,46]. In the TRIDENT-1 study, no on-target *ROS1* resistance mutations were identified among repotrectinib-treated patients who progressed in the ROS1 TKI-naïve patient population [19]. However, a comparative analysis of resistance mutations among progressors was not carried out in the current study due to limited reporting. Nevertheless, the avoidance of such mutations may be related to the improvements in PFS associated with repotrectinib.

While this study represents the first set of indirect comparisons between repotrectinib and earlier generation ROS1 TKIs, there are similarities with earlier analyses. For example, the finding that HR estimates for PFS between repotrectinib and both entrectinib and crizotinib were similar to one another in the current study (both estimating around half the risk of PFS relative to repotrectinib) is consistent with other indirect comparisons that evaluated entrectinib vs. crizotinib, showing both agents to carry a similar risk of PFS [47,48]. Furthermore, the absolute ORR and PFS for crizotinib, as captured in the current study, are broadly consistent with (and slightly better than) a recent systematic review of the real-world effectiveness of crizotinib, indicating that the current trial-based analyses may generalize to real-world settings [49].

### 4.2. Safety

Consistent with previous reports, safety with ROS1 TKIs was manageable, and no new safety signals were detected. Discussions between health care providers and patients regarding toxicity profiles should occur prior to initiating the administration of a systemic drug.

### 4.3. Study Strengths and Limitations

The systematic literature review and population-adjusted comparisons were conducted and reported in alignment with good practice guidance [23,36,50,51]. The prognostic and effect modifying factors were identified based on an a priori set of factors; SAs and residual systematic bias were assessed, and there were no patients with highly influential weights in the analysis, unduly influencing outcomes.

The primary limitation is the use of indirect methods for relative effect estimation. This was unavoidable as relevant RCTs in *ROS1+* aNSCLC are not yet available (though they may be forthcoming) [52,53]. Nevertheless, unanchored MAICs rely on strong assumptions and carry a high risk of bias, even when well executed [54]. One aspect of validity requires that the model is accurately adjusted for all prognostic factors and effect modifiers [36]. In the current analysis, several known prognostic factors could not be included in the MAIC due to the study design (e.g., ECOG PS 2), non-reporting (e.g., TP53 mutation status; baseline brain metastasis), or outcome definitions (e.g., investigator- vs. BICR-based). Furthermore, the number of prior lines of systemic therapy (which was considered weakly predictive of outcomes for ROS1 TKIs a priori) was omitted from the base case MAIC vs. crizotinib due to large imbalances. However, SAs and residual bias assessments suggested that the study’s conclusions were robust to these omissions.

The relatively small sample size of the TRIDENT-1 EXP-1 cohort was a limitation, especially given precision after adjustment in terms of TRIDENT-1 ESS. The sample sizes in the trials contributing to the comparator populations were also of concern; however, by pooling the comparator trials, the full evidence base could be incorporated into the analysis to retain an appropriate sample size. Future adaptations of population adjustment methods to allow for multiple single-arm and non-comparative studies may provide methodological improvements for combining comparator trials into population-adjusted evidence synthesis [36,54,55].

## 5. Conclusions

This analysis demonstrated a strong PFS benefit for repotrectinib relative to the earlier generation ROS1 TKIs crizotinib and entrectinib. The findings were robust across different supplemental analyses and supported by numerically favorable results for DoR and ORR. These results, alongside the published TRIDENT-1 clinical data, further support repotrectinib as a potential new standard of care for patients with TKI-naïve *ROS1+* aNSCLC.

## Figures and Tables

**Table 1:** Baseline characteristics used to generate MAIC weights.

Proportion of Patients	TRIDENT-1 Unweighted	Crizotinib		Entrectinib	
		TRIDENT-1 EXP-1 Weighted	All-Evidence Analysis Set	TRIDENT-1 EXP-1 Weighted	ALKA-STARTRK-1 and -2
N (ESS * for weighted)	71	62.3	277	30.1	168
Median:					
Age (years)	57	55.7	55.7	54.0	54.0
Proportion:					
CNS/brain metastasis at baseline (per BICR)	25.4	-	-	28.6	28.6
CNS/brain metastasis at baseline † (per investigator)	32.4	15.8	15.8	-	-
0 prior lines of systemic therapy	71.8	70.4 ‡	14.1	37.5	37.5
Never smoker	71.4 ¶	69.7	69.7	64.3	64.3
Asian race	67.6	70.2	70.2	46.4	46.4
Female	60.6	59.3	59.3	65.5	65.5
ECOG PS 0	33.8	35.3	35.3	39.3	39.3

Abbreviations: BICR—Blinded Independent Central Review; CNS—central nervous system; ECOG PS—Eastern Cooperative Oncology Group performance status; ESS—effective sample size. Note: Hazard ratios < 1 favor repotrectinib. * ESS was approximated as the square of the summed weights divided by the sum of the squared weights. This differs from the time-0 n-at-risk in Figure 1, which is represented by the sum of the patient weights. † CNS/brain metastasis at baseline was assessed by independent radiologic review (OO-1201 trial), unspecified (AcSé, EUCROSS, METROS trials) or BICR (TRIDENT-1). ‡ The 0 prior lines of systemic therapy variable was not adjusted for in the MAIC comparing repotrectinib to crizotinib. ¶ The percentage of never smokers differs from that reported in the baseline table of TRIDENT-1 EXP-1 due to the imputation of values for the eight patients that did not have this data collected.

**Table 2:** Unadjusted and adjusted comparisons of time-to-event endpoints, progression-free survival, and duration of response.

		TRIDENT-1		Comparator	
	Hazard Ratio (95% CI)	N or ESS *	Median (Months) (95% CI)	N	Median (Months) (95% CI)
Repotrectinib vs. crizotinib					
PFS, unadjusted	0.50 (0.34, 0.74)	71	35.7 (27.3, NE)	273 †	14.6 (12.8, 18.5)
PFS, adjusted	0.44 (0.29, 0.67)	62.3	37.1 (30.4, NE)	273	14.6 (12.8, 18.5)
DoR, unadjusted	- ‡	56	34.1 (27.4, NE)	- ‡	- ‡
DoR, adjusted	- ‡	- ‡	- ‡	- ‡	- ‡
Repotrectinib vs. entrectinib					
PFS, unadjusted	0.55 (0.37, 0.83)	71	35.7 (27.4, NE)	168	15.7 (12.0, 21.1)
PFS, adjusted	0.57 (0.36, 0.91)	30.1	31.1 (24.6, NE)	168	15.7 (12.0, 21.1)
DoR, unadjusted	0.57 (0.35, 0.94)	56	34.1 (27.4, NE)	114	20.5 (14.8, 34.8)
DoR, adjusted	0.60 (0.35, 1.05)	21.5	33.6 (27.4, NE)	114	20.5 (14.8, 34.8)

Abbreviations: CI—confidence interval; DoR—duration of response; ESS—effective sample size; NE—not estimable; PFS—progression-free survival. Note: Hazard ratios < 1 favor repotrectinib. * N for comparator trials and unadjusted analyses; ESS for adjusted analyses after weighting the TRIDENT-1 EXP-1 IPD. † Four patients enrolled in EUCROSS and included in baseline reporting were found to have major protocol violations and were not included in the efficacy population. ‡ PseudoIPD could not be generated for the crizotinib All-evidence analysis set due to non-reporting of DoR Kaplan–Meier curves in the publications of crizotinib studies; formal adjusted comparison using MAIC methodology was not conducted.

**Table 3:** Unadjusted and adjusted comparisons of objective response rates.

		TRIDENT-1		Comparator	
	Odds Ratio (95% CI)	N or ESS *	% ORR (95% CI)	N	% ORR (95% CI)
Repotrectinib vs. crizotinib					
ORR, unadjusted	1.56 (0.83, 2.91)	71	79 (69, 88)	272 †	71 (65, 76)
ORR, adjusted	1.48 (0.76, 2.87)	62.3	78 (68, 88)	272 †	71 (65, 76)
Repotrectinib vs. entrectinib					
ORR, unadjusted	1.77 (0.92, 3.41)	71	79 (69, 88)	168	68 (60, 75)
ORR, adjusted	1.53 (0.63, 3.66)	30.1	76 (62, 91)	168	68 (60, 75)

Abbreviations: CI—confidence interval; ESS—effective sample size; ORR—objective response rate. Note: Odds ratios > 1 favor repotrectinib. * N for unadjusted analyses; ESS for adjusted analyses after weighting the TRIDENT-1 EXP-1 IPD. † One patient in the AcSé study was not considered for ORR analysis due to an early and accidental death. This patient was included in the PFS analysis.

## Data Availability

BMS policy on data sharing may be found at https://www.bms.com/researchers-and-partners/clinical-trialsand-research/disclosure-commitment.html (accessed on 8 January 2025).

## Supplementary Materials

The following supporting information can be downloaded at https://www.mdpi.com/article/10.3390/cancers17050748/s1: Table S1: PICOS criteria for inclusion in the systematic literature review; Table S2: MAIC model specification for TRIDENT-1 EXP-1 vs. crizotinib, All-evidence analysis set; Table S3: MAIC model specification for TRIDENT-1 EXP-1 vs. pooled entrectinib trials; Figure S1: PRISMA flow diagram of studies identified in the systematic literature review; Table S4: Key design features across all studies; Table S5: Baseline characteristics for key prognostic and effect modifying factors across all studies; Table S6: Reported outcomes and outcome definitions across included trials; Appendix S1; Figure S2: Supplemental analyses of progression-free survival for repotrectinib vs. crizotinib among TKI-naïve patients with *ROS1+* aNSCLC, All-evidence analysis set; Figure S3: Supplemental analyses of objective response rate for repotrectinib vs. crizotinib among TKI-naïve patients with *ROS1+* aNSCLC, All-evidence analysis set; Figure S4: Kaplan–Meier curves of progression-free survival for repotrectinib vs. crizotinib (PROFILE 1001 only); Figure S5: Supplemental analyses of progression-free survival for repotrectinib vs. entrectinib among TKI-naïve patients with *ROS1+* aNSCLC; Figure S6: Supplemental analyses of objective response rate for repotrectinib vs. entrectinib among TKI-naïve patients with *ROS1+* aNSCLC; Figure S7: Supplemental analyses of duration of response for repotrectinib vs. entrectinib among TKI-naïve patients with *ROS1+* aNSCLC; Table S7: Treatment-related adverse event-associated outcomes.
