# Supplementary material for: Population-Adjusted Indirect Treatment Comparisons of Repotrectinib Among Patients with ROS1+ NSCLC
Source: Cancers (Basel). 2025 Feb 22;17(5):748. doi: 10.3390/cancers17050748 (PMC11899369; doi:10.3390/cancers17050748)
Supplement: Supplementary file 1 [file cancers-17-00748-s001.zip › cancers-3443307-supplementary.pdf]

cancers MDPI

Supplementary materials

Table S1. PICOS criteria for inclusion in the systematic literature review.

PICOS	Eligibility criteria
**Patients**	Patients (18 years and older) treated with anticancer therapy for *ROS1*+ advanced (stage IIIB to IV) NSCLC
**Intervention / Comparator**	ROS1 TKIs; including, but not limited to:*<br>• Repotrectinib<br>• Crizotinib<br>• Entrectinib<br>• Lorlatinib<br>• Ceritinib<br>• Taletrectinib (AB106, DS-6051b)<br>• Zidesamtinib (NVL-520)<br>• Unectrinib (TQ-B3101)<br>• Brigatinib (ARRY-162, ARRY-438162, MEK162)<br>• Cabozantinib (XL184, BMS907351)<br>Other TKIs; including:<br>• Alectinib<br>• Nintendanib + docetaxel<br>• Binimetinib (AP26113)<br>• Ensartinib (X-396)<br>• Envonalkib (TQ-B3139)<br>• Iruplinalkib<br>Platinum-based chemotherapy†<br>IOs†<br>Chemoimmunotherapy†
**Outcomes**	Survival outcomes:<br>• PFS<br>• OS<br>• CNS-PFS<br>Response outcomes:<br>• ORR<br>• DoR<br>• icORR<br>• icDOR<br>• New CNS/brain metastasis among patients with no CNS/brain metastases at baseline<br>Safety outcomes:<br>• Pooled category of grade 3/4 AEs<br>• Pooled category of serious AEs<br>• Grade 5 AEs/death<br>• Specific AEs<br>HRQoL
**Study design**	Phase 1-4 clinical trials<br>Requirement of a peer-reviewed publication criterion was included post-hoc
**Language**	Studies will be limited to publications in English

Abbreviations: AE – adverse event; CNS – central nervous system; DoR – duration of response; HRQoL – health related quality of life; icDOR – intracranial duration of response; icORR –

intracranial objective response rate; IO – immunotherapy; NSCLC – non small cell lung cancer; ORR – objective response rate; OS – overall survival; PFS – progression-free survival; ROS1+ – ROS proto-oncogene 1, receptor tyrosine kinase rearrangement positive; TKI – tyrosine kinase inhibitor. * List of investigational ROS1 TKIs may not be exhaustive. † Eligible chemotherapy, IO and chemoimmunotherapy comparators were identified within the set of trials conducted in a ROS1+ population and did not encompass all chemotherapy, IO and chemoimmunotherapy trials conducted in ROS1-negative or mixed status trial populations.

Table S2. MAIC model specification for TRIDENT-1 EXP-1 vs. crizotinib, All-evidence analysis set.

Factor	CNS / brain metastasis	ECOG PS	Smoking	Age	Race	Sex	# of prior lines
Unadjusted	û	û	û	û	û	û	û
Unadjusted*	û	û	û D	û	û	û	û
Base case	ü	ü	ü I	ü	ü	ü	û
BC + Previous LOT	ü	ü	ü I	ü	ü	ü	ü
BC smoking dropped	ü	ü	ü D	ü	ü	ü	û
BC assumed never smokers	ü	ü	ü Nev	ü	ü	ü	û
BC assumed smokers	ü	ü	ü Ev	ü	ü	ü	û
BC CNS/brain mets excluded	û	ü	ü I	ü	ü	ü	û
BC race excluded	ü	ü	ü I	ü	û	ü	û
BC ECOG excluded	ü	û	ü I	ü	ü	ü	û
BC sex excluded	ü	ü	ü I	ü	ü	û	û
BC race imputed	ü	ü	ü I	ü	ü Imp	ü	û

Abbreviations: CNS – central nervous system; ECOG PS – Eastern Cooperative Oncology Group performance status; LOT – lines of therapy; SA – supplemental analysis; unadj = unadjusted model (associated estimates may also be called unweighted or crude estimates). Note: The phase 1 portion of the TRIDENT-1 trial included patients with unknown smoking status at baseline (EXP-1; n=8). The sensitivity analyses assessed the impact of different assumptions for smoking status in these patients, including imputation, assuming all patients were never smokers, and assuming all patients had a history of smokig. * Removing the patients with smoking N/R. û D – Unadjusted model that excludes patients with missing smoking status. ü D – Scenario in which patients with missing smoking status are dropped (i.e., weight = 0). ü I – Scenario in which patients with missing smoking status are imputed to the target value of the crizotinib trials. ü Nev – Supplemental analysis in which patients with missing smoking status are set to never smokers. ü Ev – Supplemental analysis in which patients with missing smoking status are set to ever smokers. ü Imp – Supplemental analysis where Asian race values are imputed for the METROS and AcSé stuies based on the reported proportion of Asian participants in the EUCROSS study (6%).

Table S3. MAIC model specification for TRIDENT-1 EXP-1 vs. pooled entrectinib trials.

Factor	CNS / brain metastasis	ECOG PS	Smoking	Age	Race	Sex	# of prior lines
Unadjusted	û	û	û	û	û	û	û
Unadjusted*	û	û	û D	û	û	û	û
Base case	ü	ü	ü I	ü	ü	ü	ü
BC smoking dropped	ü	ü	ü D	ü	ü	ü	ü
BC assumed never smokers	ü	ü	ü Nev	ü	ü	ü	ü
BC assumed smokers	ü	ü	ü Ev	ü	ü	ü	ü
BC CNS/brain mets excluded	û	ü	ü I	ü	ü	ü	ü
BC previous LoT excluded	ü	ü	ü I	ü	ü	ü	û
BC race excluded	ü	ü	ü I	ü	û	ü	ü
BC age as categorical	ü	ü	ü I	üCat	ü	ü	ü
BC ECOG excluded	ü	û	ü I	ü	ü	ü	ü
BC sex excluded	ü	ü	ü I	ü	ü	û	ü

Abbreviations: CNS – central nervous system; ECOG PS – Eastern Cooperative Oncology Group performance status; SA – supplemental analysis. Note: The phase 1 portion of the TRIDENT-1 trial included patients with unknown smoking status at baseline (EXP-1; n=8). The sensitivity analyses assessed the impact of different assumptions for smoking status in these patients, including

imputation, assuming all patients were never smokers, and assuming all patients had a history of smokig. * Removing the patients with smoking N/R. û D – Unadjusted model that excludes patients with missing smoking status. ü D – Scenario in which patients with missing smoking status are dropped (i.e., weight = 0). ü I – Scenario in which patients with missing smoking status are imputed to the target value of the entrectinib trial. ü Nev - Supplemental analysis in which patients with missing smoking status are set to never smokers. ü Ev - Supplemental analysis in which patients with missing smoking status are set to ever smokers. ü Cat – Supplemental analysis where age is coded as a categorical variable.

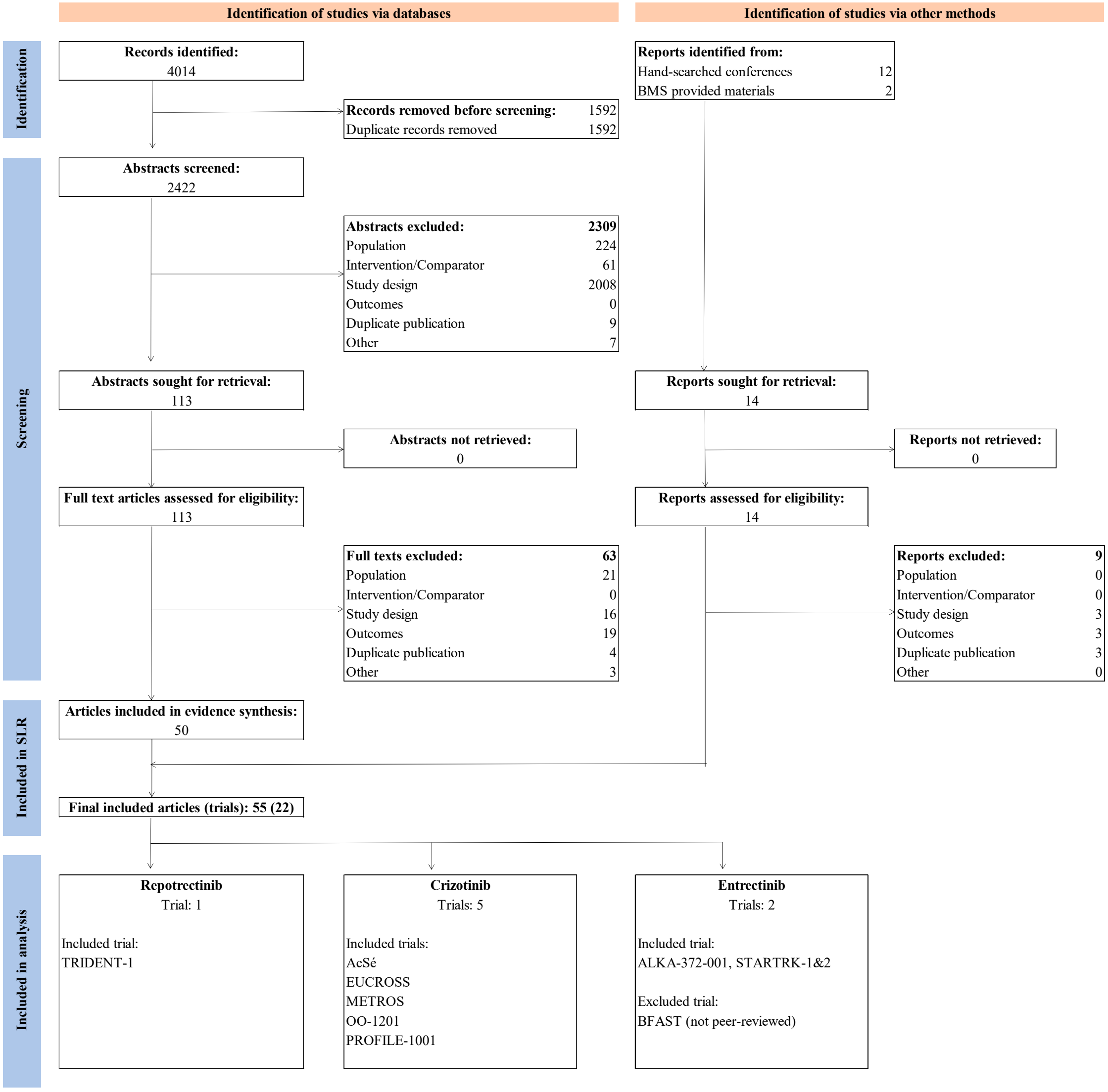

Figure S1. PRISMA flow diagram of studies identified in the systematic literature review.

Table S4. Key design features across all studies.

Study name, Cohort	Design elements			Inclusion criteria				Exclusion criteria	
	Study design	Study location(s)	Age (years)	Diagnosis	ECOG PS	Tumor assessment	Previous treatment	Previous treatment	CNS/brain metastases
TRIDENT-1, EXP-1[19]	Phase 1/2 non-randomized clinical trial	Asia, Australia, Europe and UK, North America*	≥12	Stage III or IV *ROS1*+ NSCLC	0-1	RECIST v1.1	≤1 previous chemo and/or IO†	ROS1 TKI	Symptomatic

Study name, Cohort	Design elements			Inclusion criteria				Exclusion criteria	
	Study design	Study location(s)	Age (years)	Diagnosis	ECOG PS	Tumor assessment	Previous treatment	Previous treatment	CNS/brain metastases
AcSé, *ROS1*[39]	Phase 2 non-randomized clinical trial	France	≥1	Stage III or IV *ROS1*+, *ALK*- NSCLC	0-2	RECIST v1.1	Allowed prior EFGR TKI and/or chemo	Previous crizotinib treatment	Untreated, symptomatic
EUCROSS[42,33]	Phase 2 non-randomized clinical trial	Germany, Spain, Switzerland	≥18	Stage III or IV *ROS1*+ NSCLC	0-2	RECIST v1.1	-	Any prior ROS1/ALK TKI	Symptomatic
METROS, Cohort A[41]	Phase 2 non-randomized clinical trial	Italy	≥18	Stage III or IV *ROS1*+ NSCLC	0-2	RECIST v1.1	≥1 previous chemo line	-	Symptomatic
OO-1201[38]	Phase 2 non-randomized clinical trial	East Asia	≥18	Stage III or IV *ROS1*+, *ALK*- NSCLC	0-1	RECIST v1.1	-	>3 previous lines of systemic therapies for advanced disease	Symptomatic
PROFILE 1001, *ROS1* expansion[13]	Phase 1 non-randomized clinical trial expansion cohort	Japan, Korea, and US	≥18	Stage III or IV *ROS1*+ NSCLC	0-1‡	RECIST v1.0¶	-	-	Untreated; neurologic-ally unstable
ALKA-STARTRK-1 &-2, TKI naïve cohort[14]	Pooled analysis of phase 1 and 2 non-randomized clinical trials	Asia, Australia, Europe and UK, US#	≥18	Stage III or IV *ROS1*+ NSCLC	0-2; life expec-tancy ≥3 months§	Measurable by RECIST 1.1	Previous chemo and/or IO allowed	ROS1 TKI**	Not specified

Abbreviations: ALK – anaplastic lymphoma kinase; chemo – chemotherapy; CNS – central nervous system; ECOG PS – Eastern Cooperative Oncology Group performance status; IO – immunotherapy; NR – not reported; NSCLC – non-small cell lung cancer; P – phase; RECIST – Response Evaluation Criteria in Solid Tumors; *ROS1*+ – *ROS* 1 proto-oncogene positive; TKI – tyrosine kinase inhibitor; UK – United Kingdom; US – United States. * TRIDENT-1 EXP-1 patients were enrolled from Asia (China, Hong Kong, Republic of Korea, Singapore); Australia; Canada; Europe (Spain, France, Italy, Poland) and UK; and US. † Total of 4 patients in TRIDENT-1 had two or more prior lines of therapy despite eligibility criterion. ‡ Those with an ECOG status of 2 could be enrolled into the study upon agreement between the investigator and sponsor. ¶ RECIST v1.1 for the three patients in the ALK-negative aNSCLC cohort. # In the entrectinib trial, patients were recruited from: Asia (China, South Korea, Hong Kong, Japan, Singapore, Republic of Taiwan); Australia, Europe (Italy, Spain, Belgium, France, Germany, Netherlands, Poland) and UK; and US. § ≥4 weeks in STARTRK-2. ** Previous TKI not allowed in ALKA & STRTRK-1 but allowed in STRTRK-2; pooled efficacy analysis only included TKI naïve patients; safety analysis included both TKI naïve and experienced.

Table S5. Baseline characteristics for key prognostic and effect modifying factors across all studies.

	**TRIDENT-1, EXP-1**	**AcSé, *ROS1***	**EUCROSS**	**METROS, Cohort A**	**OO-1201**	**PROFILE 1001, *ROS1* expansion**	**ALKA-STARTRK-1 &-2, TKI naive**
	N=71	N=37	N=34	N=26	N=127	N=53	N=168
% CNS/brain metastasis at baseline							
Per BICR	18 (25.4)	-	-		23 (18.1)¶	-	48 (28.6)
Per investigator	23 (32.4)	-	-	-	-	-	58 (34.5)
Measurable	8 (11.3)	-	-	-	23 (18.1)	-	25 (14.9)
Unspecified	-	8 (21.6)	7 (20.6)	6 (23.1)	-	-	-
TP53 mutation	-	-	5 (14.7)	-	-	-	-
ECOG PS							
0	24 (33.8)	11 (29.7)	12 (35.3)	18 (69.2)	34 (26.8)	23 (43.4)	66 (39.3)
1	47 (66.2)	16 (43.2)	20 (58.8)	7 (26.9)	93 (73.2)	29 (54.7)	86 (51.2)
2	0 (not eligible)	9 (24.3)	2 (5.9)	1 (3.8)	0 (not eligible)	1 (1.9%)	16 (9.5)
Smoking status							
Previous or current smoker	18 (25.4)	11 (29.7)	11 (32.4)	12 (46.2)	36 (28.3)	13 (24.5)	60 (35.7)
Never smoker	45 (63.4)	26 (70.3)	23 (67.7)	14 (53.8)	91 (71.1)	40 (75.5)	108 (64.3)
Not collected	8 (11.3)	-	-	-	-	-	-
Age							
Median (range)	57 (28, 80)	62 (33, 81)	56 (33, 84)	68 (28, 86)	62 (33, 81)	55 (25, 81)	54 (20, 86)
<65 years	52 (73)	-	-	-	106 (83.5)	38 (72.0)	-
≥65 years	19 (27)	-	-	-	21 (16.5)	15 (28.0)	-
Race							
Asian	48 (67.6)	-	2 (5.9)	-	127 (100.0)	21 (39.6)	78 (46.4)
White	18 (25.4)	-	31 (91.2)	-	0 (0.0)	30 (56.6)	72 (42.9)
Black or African American	1 (1.4)	-	0 (0.0)	-	0 (0.0)	2 (3.8)	8 (4.8)
Other	1 (1.4)	-	1 (2.9)	-	0 (0.0)	0 (0.0)	2 (1.2)
Not reported	3 (4.2)	-	0 (0.0)	-	0 (0.0)	0 (0.0)	8 (4.8)
Region							
Asia	41 (57.7)	-	-	-	127 (100.0)	-	-
United States	11 (15.5)	-	-	-	0 (0.0)	-	-
Europe, Canada, Australia	19 (26.8)	-	-	-	0 (0.0)	-	-
Sex							
Female	43 (60.6)	26 (70.3)	19 (55.9)	16 (61.5)	73 (57.5)	30 (56.6)	110 (65.5)
Male	28 (39.4)	11 (29.7)	15 (44.1)	10 (38.5)	54 (42.5)	23 (43.4)	58 (34.5)
Prior lines of systemic therapy*							
0	51 (71.8)	1 (2.7)	7 (20.6)	0 (0.0)	24 (18.9)	7 (13.2)	63 (37.5)
1	16 (22.5)	36 (97.3)†	12 (35.3)	20 (76.9)	53 (41.7)	22 (41.5)	65 (38.7)
≥2	4 (5.6)‡	-	15 (44.1)	6 (23.1)	50 (39.4)	24 (45.3)	40 (23.8)

Abbreviations: BICR – Blinded Independent Central Review; CNS – central nervous system; ECOG PS – Eastern Cooperative Oncology Group performance status; NR – not reported. * Non-ROS1 TKIs. † ≥1 previous line of non-ROS1 TKI therapy. ‡ In TRIDENT-1 EXP-1, 2 patients had 2 prior lines of therapy, and 2 patients had 3 or more prior lines of therapy. ¶ Baseline screening assessments reviewed by independent radiology laboratory.

Table S6. Reported outcomes and outcome definitions across included trials.

Trial, Cohort	Outcome definition	N	Estimate	95% CI	Data cut-off
	**Progression-free survival**				
TRIDENT-1, EXP-1*	The time from the first dose to first documented radiographic PD or death from any cause and measured by BICR using RECIST v1.1.	71	35.7	27.3, NE	15-Oct-23
AcSé, *ROS1*[39]	The interval between date of registration and the day of first documented sign of disease progression, day of death or last follow up. Tumor assessments were performed by central review, using RECIST v1.1 criteria.†	37	5.5	4.2, 9.1	Not reported
EUCROSS[42]	The time from the therapy initiation to first documented radiographic PD or death from any cause. Patients who did not meet these criteria were censored at the date of the last examination. Tumor assessments were performed by investigators, using RECIST v1.1 criteria.	30	19.4	10.1, 31.1	1-Jan-20
METROS, Cohort A‡ [41]	The date of starting therapy to the date of first evidence of either disease progression or death of the patient in the absence of documented disease progression. Patients without an event were censored at the date of last follow-up. Tumor assessments were performed by investigators, using RECIST v1.1 criteria.	26	22.8	15.2, 30.3	30-Sep-17
OO-1201[25]	Not reported.	127	15.9	12.9, 24.0	30-Jul-16
PROFILE 1001[13]	The time from the therapy initiation to first documented radiographic PD or death from any cause. Tumor assessments were performed by investigators, using RECIST v1.0 criteria.†	53	19.3	15.2, 39.1	30-Jun-18
ALKA-STARTRK-1 &-2, TKI-naïve¶ [14]	The time from the first dose to first documented radiographic PD or death from any cause and measured by BICR using RECIST v1.1.	168	15.7	12.0, 21.1	31-Aug-20
	**Duration of response**				
TRIDENT-1, EXP-1*	The first date of objective response (either CR or PR) to first documentation of radiographic disease progression, as assessed by BICR using RECIST v1.1. The DOR will be censored at the last tumor assessment date for subjects without disease progression.	56	34.1	27.4, NE	15-Oct-23
AcSé, *ROS1*[39]	The date that the criteria of CR/PR are met for the first time and the first date of documented re-appearance of the disease (recurrence, progression or death). The patient is censored at the date of the last follow up examination. Tumor assessments were performed by central review, using RECIST v1.1 criteria.†	-	-	-	-
EUCROSS[33]	As assessed by the study investigator. # Definition not provided.	21§	19.0	8.3, NE	3-Apr-17
METROS, Cohort A‡ [41]	Not reported.	17	21.4	12.7, 30.1	30-Sep-17
OO-1201[25]	Not reported	91	19.7	14.1, NE	30-Jul-16
PROFILE 1001[13]	The first date of objective response (either CR or PR) to first documentation of radiographic disease progression or death. Tumor assessments were performed by investigators, using RECIST v1.0 criteria.†	38	24.7	15.2, 45.3	30-Jun-18
ALKA-STARTRK-1 &-2, TKI-naïve¶ [14]	The first date of objective response (either CR or PR) to first documentation of radiographic disease progression or death, as assessed by BICR using RECIST v1.1.	114	20.5	14.8, 34.8	31-Aug-20

Trial, Cohort	Outcome definition	N	Estimate	95% CI	Data cut-off
	**Objective response rate**				
TRIDENT-1, EXP-1*	The proportion of subjects with a confirmed CR or PR, as assessed by BICR using RECIST v1.1.	71	79	68, 88	15-Oct-23
AcSé, *ROS1*** [39]	The best response over the first six months, as assessed by central review using RECIST v1.1.	36	69.4	53, 82	Not reported
EUCROSS[42]	The proportion of subjects with a confirmed CR or PR, as assessed by the study investigator‡ using RECIST v1.1.	30	70	50.6, 85.3	1-Jan-20
METROS, Cohort A‡ [41]	The proportion of subjects with a confirmed CR or PR, as assessed by investigator using RECIST v1.1.	26	65	44, 82	30-Sep-17
OO-1201[25]	The proportion of subjects with a best overall response of a confirmed CR or PR, as assessed by IRR using RECIST v1.1.	127	71.7	63.0, 79.3	30-Jul-16
PROFILE 1001[13]	The proportion of patients with a best overall response of confirmed CR or PR from investigator assessment using RECIST 1.0 criteria.	53	72	58, 83	30-Jun-18
ALKA-STARTRK-1 &-2, TKI-naïve¶ [14]	The proportion of subjects with a confirmed CR or PR, as assessed by BICR using RECIST v1.1.	168	67.9	60.2, 74.8	31-Aug-20

Abbreviations: CI – confidence interval; CR – complete response; IRR – independent radiologic review; NE – not estimable; PR – partial response. * Data from the Oct2023 DCO. † Response definition as reported for ORR; assumed to be the same for PFS. ‡ Capuzzo et al. 2022[40] reported a larger sample size (n=64) and more mature data (cut-off: February 2022); however, baseline characteristic reporting was insufficient and data was used fromt eh earlier Landi pulication.[41]. ¶ Fan et al. 2022[18] reported a slightly larger sample size (n=172) and more mature data (cut-off: August 2, 2021); however, data were insufficient for several of the endpoint-specific analyses and data was used from the earlier Drilon publication[14].. # Independent radiologic review assessed results are also available. § Number of reponders based on ORR response; number of patients included in DoR analysis not explicitly reported.[33]. ** ORR in the AcSé trial was reported after 2 cycles of crizotinib; therefore, the best observed response was used for analysis and reporting.

Appendix S1

All SAs were broadly consistent with the main analyses, showing a consistent direction of effect for repotrectinib compared to crizotinib (Supplemental Figure S2 and Supplemental Figure S3). Notably, when the ‘previous lines of therapy’ variable was included as an adjustment factor in the MAIC, the effective sample size was reduced to 22.7; however, the magnitude and direction of PFS HR and ORR OR were consistent with the base case findings (MAIC HR = 0.40; 95% CI: 0.22, 0.74; MAIC OR = 1.22; 95% CI: 0.47, 3.16). Sensitivity analyses testing assumptions around the proportion of patients with baseline brain metastasis (given the non-reporting in PROFILE 1001) were consistent with the base case estimates.

When limiting the crizotinib efficacy to evidence from PROFILE 1001 (n=53), repotrectinib was numerically favored relative to crizotinib with respect to PFS but the estimate was associated with considerable uncertainty and the 95% CI spanned the null value (MAIC HR = 0.72; 95% CI: 0.38, 1.36; Supplemental Figure S4). Similarly, for ORR, there was considerable uncertainty and 95% CIs spanned the null value (MAIC OR = 0.84; 95% CI 0.31, 2.27).

Across all analyses comparing repotrectinib to entrectinib, the SAs were largely consistent with the base case analysis, and maintained the same direction of effect (Supplemental Figure 5, Supplemental Figure 6 and Supplemental Figure 7).

Analysis	PFS HR (95% CI)
Unadjusted	0.50 (0.34, 0.74)
Unadjusted*	0.47 (0.31, 0.72)
BC	0.44 (0.29, 0.67)
BC + previous LoT	0.40 (0.22, 0.74)
BC smoking dropped	0.41 (0.26, 0.65)
BC assumed never smokers	0.42 (0.28, 0.65)
BC assumed smokers	0.45 (0.30, 0.69)
BC CNS/brain mets excluded	0.49 (0.33, 0.71)
BC race excluded	0.46 (0.30, 0.70)
BC ECOG excluded	0.44 (0.29, 0.67)
BC sex excluded	0.45 (0.29, 0.68)
BC race imputed#	0.50 (0.32, 0.78)

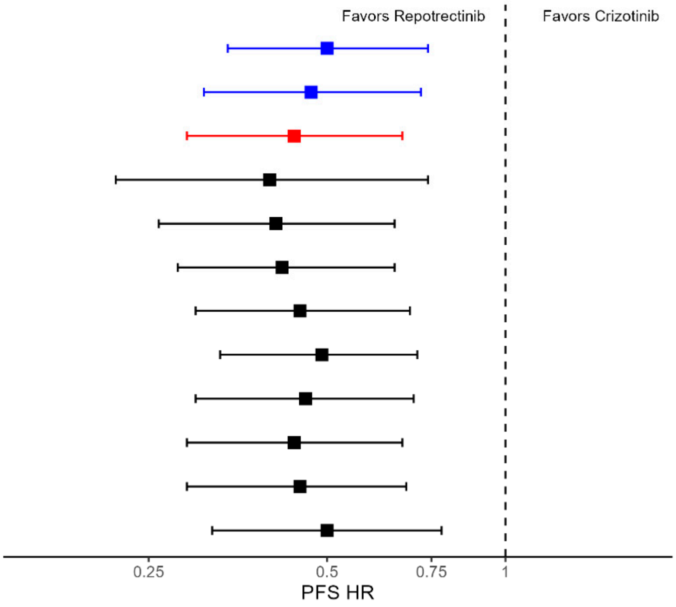

Figure S2. Supplemental analyses of progression-free survival for repotrectinib vs. crizotinib among TKI-naïve patients with *ROS1*+ aNSCLC, All-evidence analysis set. Abbreviations: BC – base case; CI – confidence interval; CNS – central nervous system; ECOG – Eastern Cooperative Oncology Group performand status; HR – hazard ratio; LOT – lines of therapy; PFS – progression-free survival. * Removing the n=8 patients with smoking not reported. # Asian race imputed for METROS and AcSé trials. Notes: The base case included all identified prognostic/effecy modifying factors available for analysis with the exception of prior lines of therapy, and imputed the smoking status of the patients in TRIDENT-1 EXP-1 who did not have smoking status reported. .

Analysis	ORR OR (95% CI)
Unadjusted	1.56 (0.83, 2.91)
Unadjusted*	1.46 (0.76, 2.79)
BC	1.48 (0.76, 2.87)
BC + previous LoT	1.22 (0.47, 3.16)
BC smoking dropped	1.46 (0.73, 2.91)
BC assumed never smokers	1.55 (0.80, 3.02)
BC assumed smokers	1.33 (0.69, 2.59)
BC CNS/brain mets excluded	1.61 (0.85, 3.04)
BC race excluded	1.42 (0.73, 2.74)
BC ECOG excluded	1.48 (0.76, 2.87)
BC sex excluded	1.45 (0.75, 2.82)
BC race imputed#	1.28 (0.65, 2.54)

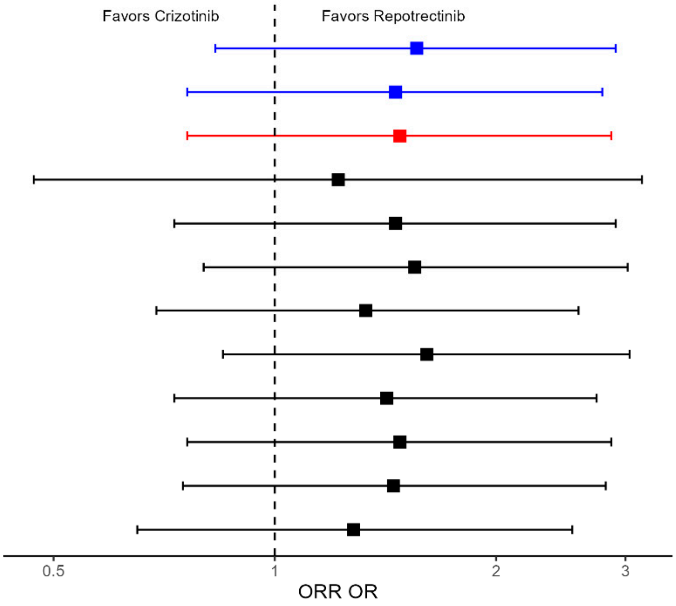

Figure S3. Supplemental analyses of objective response rate for repotrectinib vs. crizotinib among TKI-naïve patients with *ROS1*+ aNSCLC, All-evidence analysis set. Abbreviations: BC – base case; CI – confidence interval; CNS – central nervous system; ECOG – Eastern Cooperative Oncology Group performand status; LOT – lines of therapy; OR – odds ratio; ORR – objective response rate. * Removing the n=8 patients with smoking not reported. # Asian race imputed for METROS and AcSé trials. Notes: The base case included all identified prognostic/effecy modifying factors available for analysis with the exception of prior lines of

therapy, and imputed the smoking status of the patients in TRIDENT-1 EXP-1 who did not have smoking status reported. .

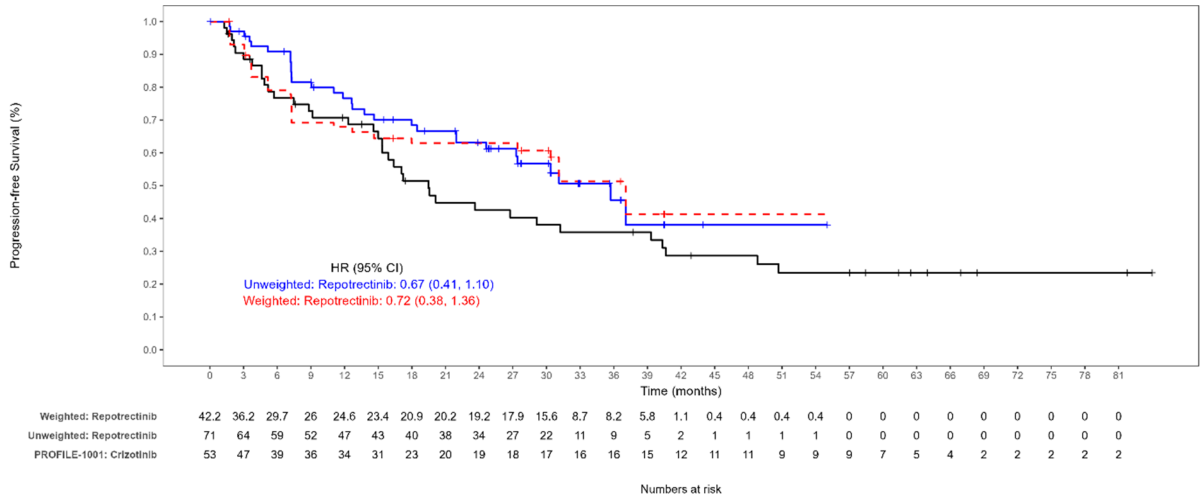

Figure S4. Kaplan-Meier curves of progression-free survival for repotrectinib vs. crizotinib (PROFILE 1001 only). Note: The weighted n-at-risk is represented by the sum of the patient weights, and is not equal to the ESS.

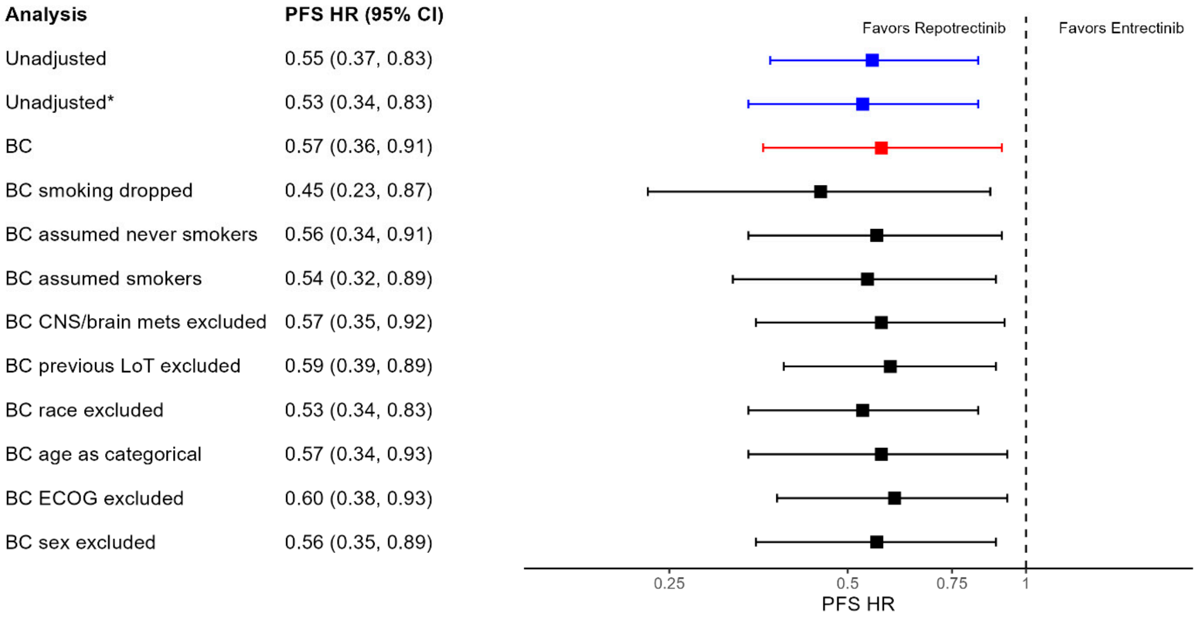

Figure S5. Supplemental analyses of progression-free survival for repotrectinib vs. entrectinib among TKI-naïve patients with ROS1+ aNSCLC. Abbreviations: BC – base case; CI – confidence interval; CNS – central nervous system; ECOG – Eastern Cooperative Oncology Group performand status; HR – hazard ratio; LoT – lines of therapy; PFS – progression-free survival. * Removing the n=8 patients with smoking not reported. Notes: The base case included all identified prognostic/effecy modifying factors available for analysis and imputed the smoking status of the patients in TRIDENT-1 EXP-1 who did not have smoking status reported. .

Analysis	ORR OR (95% CI)
Unadjusted	1.77 (0.92, 3.41)
Unadjusted*	1.66 (0.84, 3.26)
BC	1.53 (0.63, 3.66)
BC smoking dropped	1.07 (0.38, 3.00)
BC assumed never smokers	1.54 (0.60, 3.95)
BC assumed smokers	1.30 (0.52, 3.20)
BC CNS/brain mets excluded	1.37 (0.58, 3.28)
BC previous LoT excluded	1.82 (0.89, 3.73)
BC race excluded	1.63 (0.72, 3.71)
BC age as categorical	1.46 (0.58, 3.69)
BC ECOG excluded	1.44 (0.59, 3.52)
BC sex excluded	1.59 (0.65, 3.88)

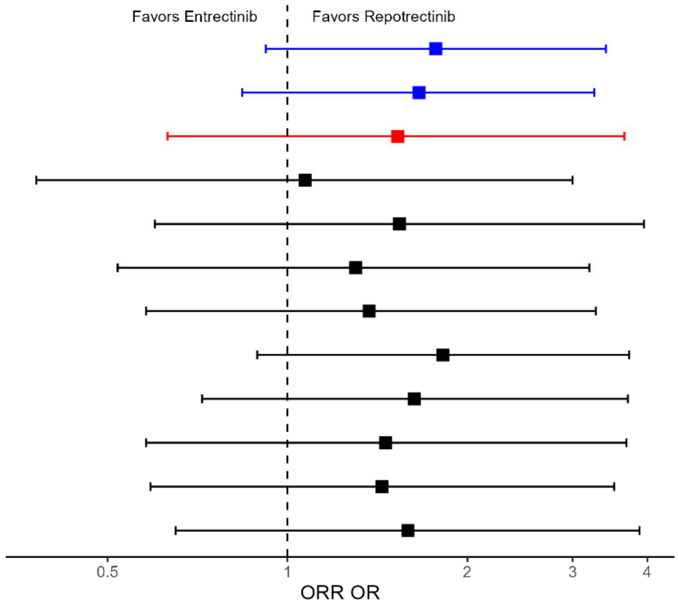

Figure S6. Supplemental analyses of objective response rate for repotrectinib vs. entrectinib among TKI-naïve patients with ROS1+ aNSCLC. Abbreviations: BC – base case; CI – confidence interval; CNS – central nervous system; ECOG – Eastern Cooperative Oncology Group performand status; LoT – lines of therapy; OR – odds ratio; ORR objective response rate. * Removing the n=8 patients with smoking not reported.Notes: The base case included all identified prognostic/effecy modifying factors available for analysis and imputed the smoking status of the patients in TRIDENT-1 EXP-1 who did not have smoking status reported. .

Analysis	DoR HR (95% CI)
Unadjusted	0.57 (0.35, 0.94)
Unadjusted*	0.51 (0.29, 0.89)
BC	0.60 (0.35, 1.05)
BC smoking dropped	0.37 (0.16, 0.84)
BC assumed never smokers	0.58 (0.32, 1.02)
BC assumed smokers	0.56 (0.30, 1.01)
BC CNS/brain mets excluded	0.56 (0.31, 1.01)
BC previous LoT excluded	0.59 (0.36, 0.98)
BC race excluded	0.61 (0.35, 1.06)
BC age as categorical	0.56 (0.31, 1.02)
BC ECOG excluded	0.64 (0.37, 1.09)
BC sex excluded	0.60 (0.35, 1.04)

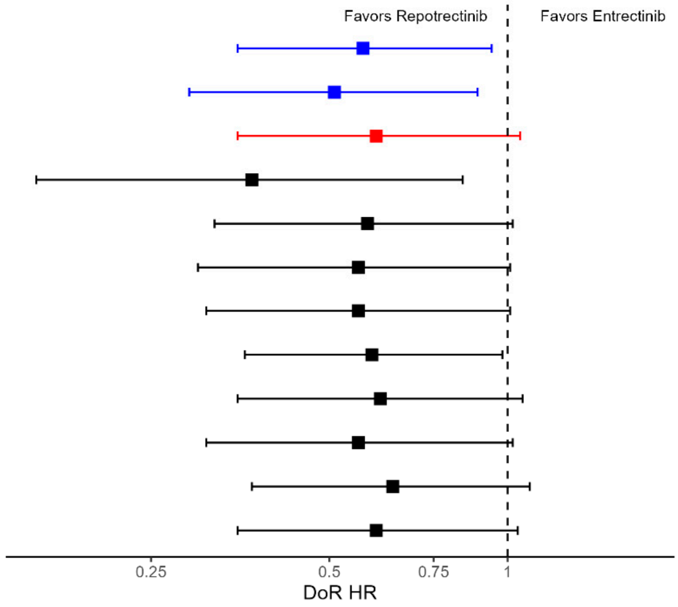

Figure S7. Supplemental analyses of duration of response for repotrectinib vs. entrectinib among TKI-naïve patients with *ROS1+* aNSCLC. Abbreviations: BC – base case; CI – confidence interval; CNS – central nervous system; DoR – duration of response; ECOG – Eastern Cooperative Oncology Group performand status; HR – hazard ratio; LoT – lines of therapy. * Removing the n=8 patients with smoking not reported. Notes: The base case included all identified prognostic/effecy modifying factors available for analysis and imputed the smoking status of the patients in TRIDENT-1 EXP-1 who did not have smoking status reported.

Table S7: Treatment related adverse events associated outcomes.

Trial name, Cohort	N	Treatment related adverse events					
		Permanent discontinuation n (%)	Treatment interruption n (%)	Dose modification n (%)	Any grade 3/4 n (%)	Serious AEs n (%)	Deaths n (%)
TRIDENT-1*[56]	367	17 (4.6)	128 (34.9)	123 (33.5)	107 (29.2)	29 (7.9)	1 (<0.1)
ALKA-STARTRK-1&-2†[18]	247	17 (6.9)	89 (36.0)	86 (34.8)	107 (43.3)	35 (14.2)	1 (0.4)
AcSé, *ROS1*[39]	35	3 (8.6)	-	-	-	-	-
EUCROSS[33]	34	3 (8.8)	17 (50.0)	18 (52.9)	8 (23.5)	5 (14.7)	1 (2.9)
OO-1201[38, 57]	127	3 (2.4)	39 (30.7)	22 (17.3)	41 (32.3)	11 (8.7)	1 (0.8)
METROS, Cohort A[41]	26	-	-	-	8 (31.0)	0 (0.0)	0 (0.0)
PROFILE 1001, TKI-naive[13]	53	0 (0.0)	-	-	19 (35.9)	-	-

Abbreviations: AE – adverse events; *ROS1* – *ROS* proto-oncogene 1, receptor tyrosine kinase rearrangement; TKI – tyrosine kinase inhibitor; TRAE – treatment-related adverse event. * Safety population for TRIDENT-1 were pooled across all ROS1+ NSCLC cohorts, including both ROS1 TKI-naïve and -experienced patients. † Pooled entrectinib analysis included patients who received at least one dose of entrectinib.
